# Requirements for the Packaging of Geminivirus Circular Single-Stranded DNA: Effect of DNA Length and Coat Protein Sequence

**DOI:** 10.3390/v12111235

**Published:** 2020-10-30

**Authors:** Keith Saunders, Jake Richardson, David M. Lawson, George P. Lomonossoff

**Affiliations:** 1 Department of Biological Chemistry, John Innes Centre, Norwich Research Park, Norwich NR4 7UH, UK; david.lawson@jic.ac.uk (D.M.L.); george.lomonossoff@jic.ac.uk (G.P.L.); 2 Department of Cell and Developmental Biology, John Innes Centre, Norwich Research Park, Norwich NR4 7UH, UK; jake.richardson@jic.ac.uk

**Keywords:** geminivirus, coat protein, capsid morphology, encapsidation, AYVV

## Abstract

Geminivirus particles, consisting of a pair of twinned isometric structures, have one of the most distinctive capsids in the virological world. Until recently, there was little information as to how these structures are generated. To address this, we developed a system to produce capsid structures following the delivery of geminivirus coat protein and replicating circular single-stranded DNA (cssDNA) by the infiltration of gene constructs into plant leaves. The transencapsidation of cssDNA of the *Begomovirus* genus by coat protein of different geminivirus genera was shown to occur with full-length but not half-length molecules. Double capsid structures, distinct from geminate capsid structures, were also generated in this expression system. By increasing the length of the encapsidated cssDNA, triple geminate capsid structures, consisting of straight, bent and condensed forms were generated. The straight geminate triple structures generated were similar in morphology to those recorded for a potato-infecting virus from Peru. These finding demonstrate that the length of encapsidated DNA controls both the size and stability of geminivirus particles.

## 1. Introduction

The family *Geminiviridae*, named because of the characteristic twinned morphology of their particles [1], includes nine genera [2]. These are widely distributed and are responsible for many diseases of food and commodity crops worldwide [3,4,5]. The genomes of the majority of the genera consist of a single molecule of circular single-stranded DNA (cssDNA) [6], approximately 2.5–3.0 kb in size that is encapsidated within the twinned particles. However, the genomes of the *Begomovirus* genus are usually bipartite, consisting of two molecules of cssDNA that are approximately the same size (2.6–2.8 kb), each possessing an identical DNA sequence of about 200 nucleotides. This consists of a bidirectional mRNA promoter and the sequences necessary for the initiation and termination of genomic DNA synthesis [7]. However, some *Begomoviruses* lack the full-sized genomic component but are instead associated with a half-sized cssDNA (1.3–1.4 kb) of the *Tolecusatellitidae* family, a DNA entity formerly referred to as a betasatellite, which is necessary for infection of their natural host plant [8,9]. These *Begomoviruses* also harbour similar sized cssDNAs of the geminivirus-associated alphasatellites (family *Alphasatellitidae,* subfamily *Geminialphasatellitinae*) that are capable of self-replication but are not essential for the progression of the viral disease [10,11]. However, some natural tomato yellow leaf curl virus (TYLCV) plant infections, a *Begomovirus* that does not require additional components for infectivity, have been shown to harbour both alpha [12] and beta satellite DNAs [13]. Indeed, both these DNAs can be maintained, and the former can be insect transmitted from artificially inoculated TYLCV plant infections [14,15]. Recently, alphasatellite DNAs, in association with *Mastrevirus* genomic DNA, have also been isolated from plants not presenting visible symptoms of disease [16]. Bipartite *Begomovirus* infections also support the propagation of half-genomic sized defective interfering DNAs [17]. Similar-sized but recombinant DNAs, consisting of sequences derived from alpha and or beta satellites and genomic DNAs, are also found in *Begomovirus* infections [18,19]. Importantly, all cssDNAs associated with *Begomoviruses* infections are encapsidated by the viral coat protein. The spread of geminivirus disease from plant to plant, by whitefly or leafhopper transmission, is dependent upon the interaction of the virus coat protein with the viral insect vector [20]. *Begomoviruses,* for example, are vectored exclusively by the whitefly *Bemisia tabaci,* whereas leafhopper vectors are responsible for the transmission of several genera including the *Mastreviruses*, that cause diseases prevalent in maize, and the *Curtoviruses*, that are responsible for curly top disease in beet. Replication of geminiviruses was originally not thought to occur in their insect vectors but replication of tomato yellow leaf curl virus in the salivary glands of its whitefly vector has recently been reported [21]; this is believed to play a major role in the dynamic spread of tomato yellow leaf curl disease.

Geminivirus particles are formed of 110 copies of a single type of coat protein arranged in two hemispheres [22,23]. Essentially, they consist of two fused *T* = 1 capsids, each lacking a pentamer at the interface between the hemispheres [24]. Naturally occurring empty geminate virus particles have never been characterised or shown to be prevalent in plant infections. Other occasional smaller and larger variations in geminivirus capsid morphology have, however, been recorded. For example, isometric or single particles, in addition to the regular geminate particles, have been characterised in *Begomovirus* and *Mastrevirus* plant infections [25,26]. These resemble particles of the *Nanoviridae*, a plant virus family whose genomes consists of multiple different cssDNAs, approximately 1 kb in size, each encapsidated in separate isometric particles [27]. Larger triple viral capsid structures, in low abundance compared to the numerous geminate particles, have been isolated from a natural beet curly top virus (BCTV) infection of Shepherd’s Purse [28] and similar structures can be found after experimental infections of *Nicotiana benthamiana* with African cassava mosaic virus (ACMV) [29,30]. Abundant triple capsid structures, in addition to relatively few geminate particle structures, have been described for virus particles found in infected potato, that of solanum apical leaf curl virus (SALCV) [31], a disease first recorded in 1982 [32]. Unfortunately to this day, there is no DNA sequence data available regarding the virus responsible for this infection. However, transmission electron microscopy of DNA extracted from partially purified virions of SALCV, revealed the presence of circular DNAs with an estimated length of approximately 3200 nucleotides [33]. The DNA of citrus chlorotic dwarf-associated virus (CCDaV), a virus characterised in diseased citrus plants, has several geminivirus-like sequence motifs within it but, with a predicted genome size of 3640 nucleotides, it is much larger than any known geminivirus [34]; however, the CCDaV capsid morphology remains unknown. Similar DNA sequences have been assembled from DNA extracted from other virus-infected plants. Characteristically these have sequence features, for example encoding the coat protein and replication- associated functions, that suggest that they are unassigned geminiviruses [35,36,37]; and that these also have circular genomes of between 3.20 and 3.75 kb. Taken together, these observations suggest that geminviral coat proteins can encapsidate different lengths of cssDNA and can form a variety of particle structures.

The first near atomic resolution of the geminivirus capsid structure indicated that inter-capsomer contacts, mediated by N-terminal regions of coat protein differ depending on their capsid location [24]. At least three structural conformations of the coat protein, [38] can be discerned. To examine the amino acids important for the generation of geminivirus particles, we have previously developed a leaf co-infiltration system. In this, the coat protein of ageratum yellow vein virus (AYVV), was expressed transiently via the pEAQ-*HT* [39] vector, in the presence of cssDNA that was provided by the self-replicating AYVV alphasatellite (DNA-α), half the size of the genomic segment [38]. Co-infiltration experiments with wild-type coat protein demonstrated that both isometric and geminate virus particles are generated at a ratio of approximately 3:2 [38]. This differs markedly from the situation with a wild-type infection where essentially 100% of particles are geminate. Mutation of two coat protein residues (R48 and M59), either singly or in combination, involved in the interface between the two hemispheres and potential interaction with the cssDNA, resulted in the production of only isometric particles [38]. Overall, these results indicated that both the coat protein sequence and the length of the encapsidated cssDNA can influence particle morphology.

In this study we use the availability of an in planta geminivirus assembly system to explore the specificity of DNA–coat protein interactions and the effects of cssDNA length on particle morphology. This system involves co-expressing cssDNA molecules of varying lengths, each lacking the viral coat protein gene, together with geminivirus coat proteins expressed via the pEAQ-*HT* expression vector. We show that geminate, along with isometric and double particles, can be produced and that it is possible to trans-encapsidate full-length AYVV DNA, but not half-length DNA-α, by the coat proteins of two different geminiviral genera. Using a further refinement of the assembly system, involving a replicating cssDNA that itself expresses its coat protein, we demonstrate that particle morphology, such as the formation of triple particles, is a consequence of increasing the size of the replicating cssDNA.

## 2. Materials and Methods

### 2.1. Plasmids for the Expression of Geminivirus Coat Proteins

Plasmids pEAQ-*HT*-D1-AYVVCP, pEAQ-*HT-*CPR48A and pEAQ-*HT-*CPM59D, expressing wild-type and mutant versions of the AYVV coat protein have been described previously [38]. The coat protein genes of ACMV (from pBin1.3A [40]), of BCTV (from pBin1.2 [20]) and of bean yellow dwarf virus (BeYDV; from pBin-BYV1.4 [41]) were amplified by the PCR using primer pairs KS155 and KS156, KS147 and KS148 and KS153 and KS154, respectively (Table S1). Following restriction enzyme digestion with *Age*I and *Xho*I, (Table S1), and purification, the DNA fragments were inserted into the plant expression vector pEAQ-*HT* [39] via its *Age*I and *Xho*I sites to yield pEAQ-*HT*-ACMVCP, pEAQ-*HT-*BCTVCP and pEAQ-*HT*-BeYDVCP, respectively. Coat protein structural comparisons [42] were performed using the https://swissmodel.expasy.org/interactive website (Swiss Institute of Bioinformatics) with the AYVV coat protein, (6f2s.1.J), as the previously determined template structure in each case.

### 2.2. Replicating Gene Constructs

**Figure 1:**
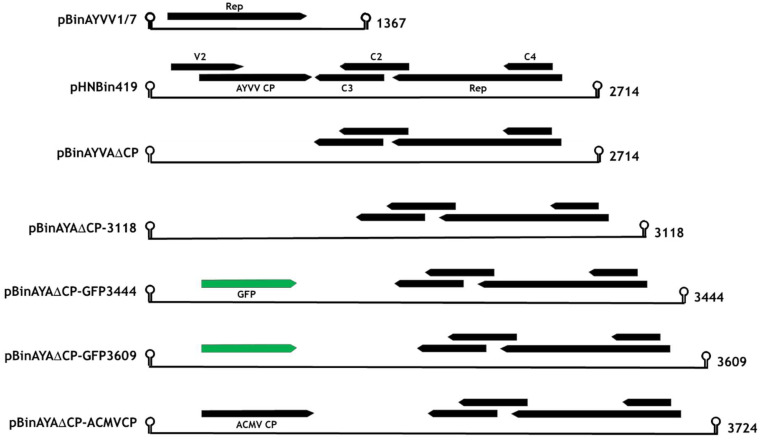
Linear representation of the replicating gene constructs with the known or predicted circular length, in nucleotides, for each displayed on the right. CP, coat protein; Rep, viral protein involved in rolling circle replication; C2, C3, C4, and V2, viral genes. GFP, green fluorescent protein.

The construction of pHNBin419, containing a partial tandem copy of the AYVV geminivirus genomic DNA, [43] and pBinAYVV1/7, containing a partial tandem copy of the AYVV alphasatellite DNA [11] have been described previously. The Agilent “QuikChange Primer Design” website, https://www.agilent.com/store/primerDesignProgram.jsp (Agilent Technologies, Santa Clara, CA, USA) was used to design primer pairs (KS133P and KS134P, Table S1), to introduce the *Age*I and *Spe*I restriction sites into the AYVV genome and to eliminate the coat protein initiating methionine (Figure S1). pHNIC419 [43] was used as the DNA template for the reaction as described in the GeneArt site-directed mutagenesis system (Invitrogen, Carlsbad, CA, USA). The sequence-verified tandem copy was transferred to pBinPlus [44] via its *Pac*I and *Asc*I restriction sites to generate pBinAYA∆CP. Plasmid pEAQ-*HT*-GFP [39] was used as a template for a series of PCR reactions with DNA primers KS138, KS154, KS144 and KS145 (Table S1), to generate DNAs that after digestion with the appropriate restriction enzymes (Table S1), were cloned into pBinAYA∆CP via its *Age*I and *Spe*I restriction sites to yield pBinAYA∆CP-3118, pBinAYA∆CP-GFP3444 and pBinAYA∆CP-GFP3609, (Figure 1). Similarly, pEAQ-*HT*-ACMVCP was used as a template for PCR amplification using primers KS 174 and KS175 (Table S1). After restriction enzyme digestion, the DNA product was cloned into pBinAYA∆CP via its *Age*I and *Spe*I restriction sites to yield pBinAYA∆CP-ACMVCP (Figure 1). DNA sequences of the coat protein expression plasmids and the replicating gene constructs were verified by DNA sequencing (Eurofins Scientific, Luxembourg).

### 2.3. Isolation of Virus-Like Particles

All plasmids were transformed in *Agrobacterium tumefaciens* strain LBA4404 and selected colonies were grown on LB medium supplemented with rifampicin and kanamycin. Following centrifugation, the bacteria were resuspended in MMA buffer (10 mM MES (2-[*N*-morpholino] ethanesulphonic acid) pH 5.6, 10 mM MgCl_2_, 100 μM Acetosyringone) to an OD_600_ of 4.0 [39] and pressure infiltrated into *N. benthamiana* leaves. Equal volumes of co-infiltrating bacteria were combined prior to infiltration. After maintenance for 7–9 days at 25 °C with supplementary lightening, the infiltrated leaves were harvested and, if necessary, photographed under natural daylight or UV light. Viral particles were extracted, purified by centrifugation through caesium sulphate gradients and dialysed as previously described [38]. Gradient fractions, collected by bottom puncture of the ultracentrifugation tube, were run in ascending order from left to right when analysed by electrophoresis, through MOPS-buffered 12% (*w/v*) NuPAGE gels (Life Technologies, Carlsbad, CA, USA). Proteins were visualised by staining with Instant Blue (Abcam, Cambridge, UK). ACMV coat protein was detected by Western blotting using a polyclonal antibody raised against ACMV particles [40] followed by detection with a goat anti-mouse secondary antibody conjugated to horseradish peroxidase and developed using the chemiluminescent substrate ECL plus (Amersham Pharmacia, Little Chalfont, UK). Western blot images were collected using the manufacturer’s software on an ImageQuant LAS 500 detector (GE Healthcare Life Sciences, Uppsala, Sweden). Viral coat protein identity was confirmed by MALDI-TOF analysis of the gel band containing the putative coat protein (JIC, Platform Facility, Norwich Research Park, UK).

### 2.4. Negative Stain Transmission Electron Microscopy

Negatively stained electron microscope (EM) grids were produced by applying 10 μL of particle preparations on to glow discharged (20 s at 10mA in an Ace 200, Leica) formvar/carbon film copper 400-mesh grids, (EM Resolutions Ltd., Sheffield, UK). After 5 min, excess sample was blotted away, and the grids were successively washed three times (each 15–20 s) by floating on water droplets and finally stained with 2% (*w/v*) uranyl acetate for 15 s. Excess solution was removed at each step by blotting with Whatman No. 1 filter paper, and the grids were air dried before examination with a Talos F200C transmission electron microscope (Thermo Fisher Scientific, Eindhoven, The Netherlands) operated at 200 kV, fitted with a OneView 4k × 4k CMOS (Gatan UK, Abingdon, Oxfordshire, UK) bottom-mounted camera (JIC Bioimaging Platform Facility). Automated data acquisition was setup using EPU, each image had a 1-s exposure with a sample dose of 40 e^−^/A^2^, nominal magnification of 45,000× and a calculated pixel size of 3.5 Å. Single particle analysis of the resultant images was performed using cryoSPARC v. 2.15.0 [45].

## 3. Results

### 3.1. Transencapsidation of Alphasatellite DNA by Geminivirus Coat Proteins

**Figure 2:**
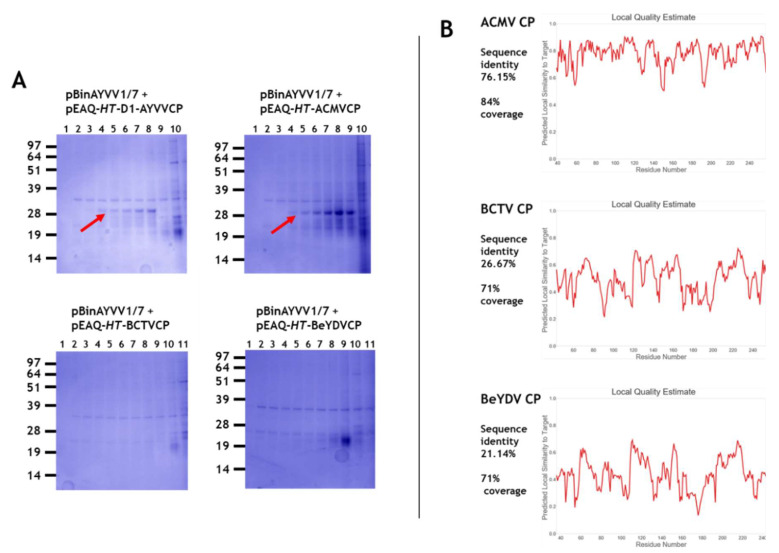
(**A**) An amount of 12% MOPS buffered NuPAGE gels of caesium sulphate gradient fractions. The infiltrated gene constructions for each gradient are indicated above each gel. Fractions are shown from the bottom (1) to the top of the gradient (10 or 11) from left to right. Red arrows indicate the position of coat protein. (**B**) Local quality estimation assessments for the 3-D relatedness of the different coat proteins to the known ageratum yellow vein virus (AYVV) coat protein 3-D structure. Relative sequence identity and structural coverage for each are indicated on the left.

Initially we wished to test whether the half-sized self-replicating AYVV alphasatellite DNA, pBinAYVV1/7 (1367 nucleotides) Figure 1, which had previously been shown to be encapsidated by AYVV coat protein [38], could interact with coat proteins from different geminivirus genera to form virus particles. The alphasatellite, was co-infiltrated with pEAQ-*HT* plasmids expressing the coat proteins from the *Begomovirus*, *Mastrevirus* and *Curtovirus* genera. Co-infiltration of pEAQ-*HT*-AYVVCP and the alphasatellite pBinAYVV1/7 acted as a positive control for the homologous assembly of coat protein into isometric and geminate capsid structures [38]. Caesium sulphate gradient centrifugation was used to analyse assembly since the coat protein associated with particles migrates to the middle of the gradient (fractions 4 to 8 or 9; arrowed in Figure 2A) while unassembled protein remains at the top. The range of buoyant densities is probably a consequence of whether one or two copies of the alphasatellite cssDNA are incorporated into the geminate particles, as well as the presence of isometric particles containing a single copy [38]. Additional host-derived proteins are also seen throughout the gradient, especially in the least dense fractions. These differ in size than those derived from the coat protein are seen in all gradients of partially purified particles. Figure 2A shows that particles are present only when the coat proteins from the members of *Begomovirus* genus, AYVV and ACMV were co-expressed with alphasatellite DNA, but not when coat proteins of two other geminivirus genera BeYDV (*Mastrevirus*) and BCTV (C*urtovirus*), were used. Electron microscope examination of the preparations of particles produced with the AYVV and ACMV coat proteins showed they contained both isometric and geminate particles (data not shown) a situation similar to that seen in Figure 3 of Hesketh et al. [38]. The occurrence of the two types of particles probably accounts for their wide distribution across the caesium gradient as they are likely to have different buoyant densities. Figure 2B illustrates 3-D coat protein relatedness, when the known 3-D structure of AYVV CP (PDB accession code 6F2S) is compared to the predicted 3-D structures of the other three geminivirus coat proteins. Relatedness when expressed as predicted local similarity [42], shows a high 76% sequence identity score between the two *Begomovirus* coat proteins, (Figure 2B upper panel). This is very much reduced when the AYVV coat protein 3-D structure is compared to the predicated 3-D structures of both the BCTV and BeYDV coat proteins, resulting in low 26% and 21% sequence identity scores, respectively (Figure 2B middle and lower panels). This structural difference may explain why the half-size cssDNA does not appear to be encapsidated by coat protein from the other geminivirus genera. However, it is also possible that encapsidation of the half-sized cssDNA was achieved by BCTV and BeYDV coat proteins, but the resulting capsids are unstable. The latter is supported by leafhopper *Circulifer tenellus* transmission experiments between *Beta vulgaris* cv. Giant Western sugar beet plants that imply that the alphasatellite cssDNA used here can be incorporated into BCTV capsids during viral infections. [46].

### 3.2. Transencapsidation of AYVV Genome-Length DNA

To investigate whether the encapsidation of full-length genomic cssDNA provides the additional protein/cssDNA interactions able to stabilise heterologous virus particles compared to half-sized cssDNA, a full-length version of the AYVV genomic DNA that cannot express the coat protein was created. The resulting construct, pBinAYA∆CP (Figure 1), designed to produce cssDNA identical in size to wild-type AYVV genomic cssDNA (2741 nucleotides) has six nucleotide changes with respect to the wild-type virus vector (Figure S1), resulting in the elimination of the initiation codon for the coat protein gene and the introduction of two restrictions sites (*Age*I and *Spe*I) immediately adjacent to this mutation (Figure S1). In addition to abolishing expression of the coat protein, the *V2* gene, associated with virus movement [7] is disrupted; however, since only infiltrated leaves are examined in the transencapsidation experiments, this is irrelevant. The restriction sites (*Age*I and *Spe*I) were used in subsequent experiments to introduce foreign DNA to increase the length of the cssDNA.

**Figure 3:**
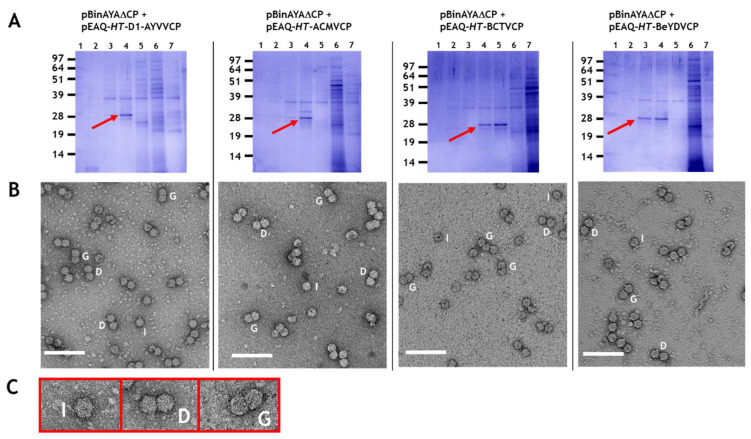
(**A**) An amount of 12% (*w/v*) MOPS-buffered NuPAGE gel resolution of caesium sulphate gradient separated virus particles. The infiltrated gene constructs for each are indicated above. Red arrows, caesium sulphate fractions that contain coat protein. (**B**) Transmission electron microscopy of caesium sulphate fractions that contain coat protein. I, isometric; D, double and G, geminate morphological virus particles. Bar = 100 nm. (**C**) Expanded images of I, D and G virus-like particles.

Plasmid pBinAYA∆CP was used in a series of co-infiltration experiments with the pEAQ-*HT* plasmids of the various wild-type geminiviral coat proteins described above to examine transencapsidation of genome-sized cssDNA. As previously stated, co-infiltration of pEAQ-*HT*-AYVVCP and pBinAYA∆CP served as a positive control for the homologous assembly of AYVV particles when the coat protein is supplied in trans. Particle preparations were analysed by centrifugation through caesium sulphate gradients. Figure 3A shows NuPAGE analysis of the resulting fractions, with the protein of the expected size of that of a geminvirus coat protein, (arrowed) appearing in fractions 4, 4 and 5 or 3 and 4 depending upon the coat protein, approximately halfway down each gradient. Mass spectrometry of tryptic digests of the protein in the bands confirmed their identity as the expected geminivirus coat proteins (Figure S2). As in previous studies, [25,47,48,49], additional faster migrating forms of the coat protein can be seen. The presence of coat protein in fractions from the middle of the gradient suggests that, unlike the encapsidation of alphasatellite DNA, all the coat proteins were able to form particles with full-length cssDNA. In addition, the coat protein was found in only one or two fractions indicating the density of the particles is more uniform than in the previous experiments. To confirm that virus particles had been produced, samples from the fractions containing the coat protein were dialysed and examined by transmission electron microscopy. Figure 3B shows that virus particles were, indeed, formed when each of the coat proteins of AYYV, ACMV, BeYDV and BCTV were co-expressed with pBinAYA∆CP. These results indicate that transencapsidation can readily occur on full-length genomic cssDNA, in contrast to results with half-length alphasatellite DNA. On close examination, many of the particles appeared to be formed of two distinct closely aligned isometric “double” particles (labelled D in Figure 3B,C) that lack the characteristic straight, full-width divisional line that separates each hemisphere in true geminate particles (labelled G). Isometric particles, labelled I, are also evident. The presence of these atypical particles occurred irrespective of the origin of the viral coat protein. They probably arise as a consequence of the lack of any coordination between viral DNA replication and coat protein synthesis that would be expected during a geminivirus infection, resulting in aberrant initiation and growth of the particles.

**Figure 4:**
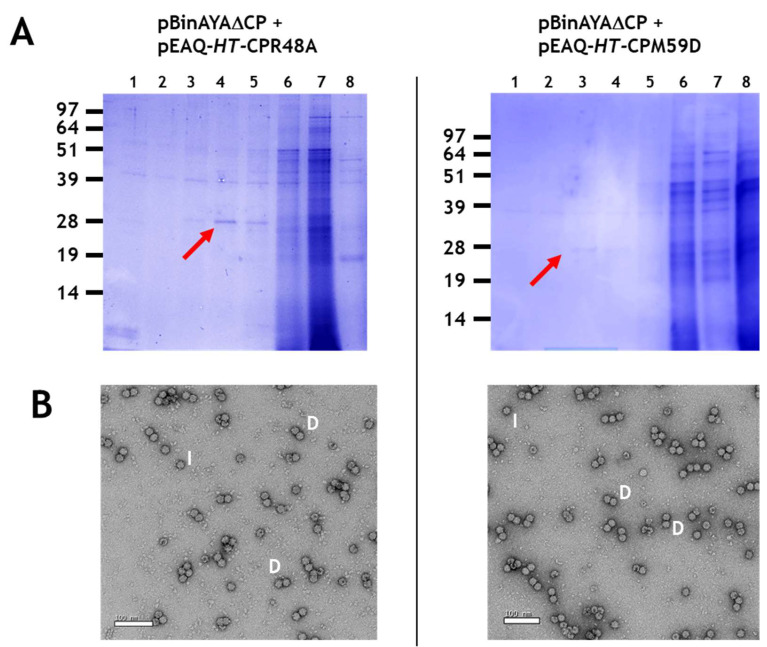
(**A**), Caesium sulphate gradient profile of protein resolved in 12% MOPS buffered NuPAGE following the co-infiltration of pBinAYA∆CP with either pEAQ-*HT*CPR48A or with pEAQ-*HT*CPM59D. Red arrows indicate caesium sulphate fractions that contain coat protein. (**B**), Transmission electron microscopy of caesium sulphate fractions that contain coat protein. I, isometric; D, double morphological particles.

The formation of double and isometric particles, as opposed to true geminate particles, was investigated further by performing co-infiltration experiments with pBinAYA∆CP and the AYVV coat protein mutants, pEAQ-*HT-*CPR48A or pEAQ-*HT-*CPM59D, that had previously been shown to be incapable of forming geminate particles with half-sized ssDNA [38]. Following caesium sulphate gradient purification (Figure 4A), NuPAGE electrophoresis showed that both mutants gave a similar gradient profile to that observed when wild-type coat protein was used, (Figure 3A). Transmission electron microscopy revealed the abundant production of only the double and isometric particles (Figure 4B), labelled D and I, respectively, and the absence of any true geminate particles. This again confirms the role of amino acids R48 and M59 in the correct formation of the equatorial region of a true geminate particle.

### 3.3. Effect on Particle Morphology of Increasing DNA Length

**Figure 5:**
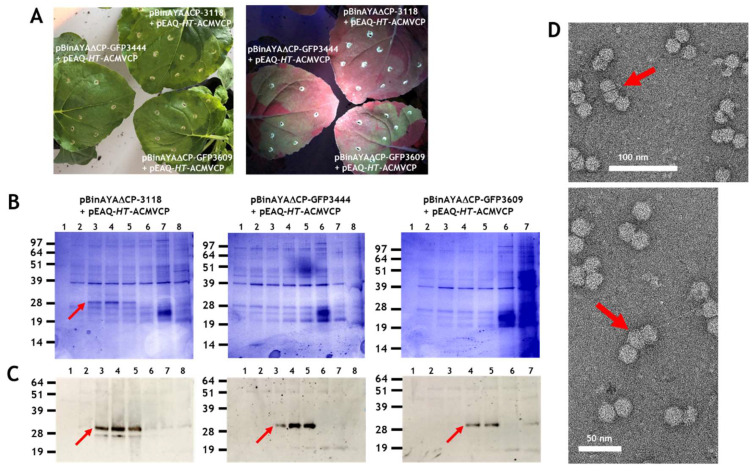
(**A**), GFP expression at 6 days post infiltration on leaves co-infiltrated with pEAQ-HT-ACMVCP and either pBinAYA∆CP-3118, or pBinAYA∆CP-GFP3444 or pBinAYA∆CP-GFP3609. Left panel photographed under natural light and right panel under UV light. (**B**), Instant Blue staining of 12% MOPS buffered NuPAGE gel of the caesium sulphate gradient profile of protein extracted from plants co-infiltrated with pEAQ-*HT*-ACMVCP and pBinAYA∆CP-3118 or with pBinAYA∆CP-GFP3444, or with pBinAYA∆CP-GFP3609 as indicated. Red arrow indicates ACMV coat protein. (**C**), ACMV coat protein (red arrow) detected by Western blotting with anti-ACMV antibody of samples resolved as in part (**B**). (**D**), Transmission electron microscopy of virus particles present in the peak fraction of the co-infiltration of pEAQ-*HT*-ACMVCP and pBinAYA∆CP3118. Red arrows indicate triple capsid structures. Bar size as indicated, 50 or 100 nm.

Particles consisting of three or more, as opposed to two substructures, have been observed during transmission electron microscopy studies of geminivirus infections and in plants transgenic for an ACMV defective-interfering DNA inoculated with ACMV [29,31]. However, the structure of these have never been characterised in detail. Larger particles potentially have the capacity to encapsidate cssDNA molecules longer than the standard genomic length (2.5–2.9 kb) found within a typical geminate particle. Longer-than-genome length replicating cssDNA molecules have been noted in studies concerning geminivirus replication [30,47,50] and it is possible that encapsidation of such molecules is associated with the formation of such larger structures [28,29,30]. To investigate the ability of longer-than-genome length cssDNAs to promote the formation of triple structures, a series of constructs based on pBinAYA∆CP was created by inserting increasing lengths of DNA derived from pEAQ-*HT*-GFP, between its *Age*I and *Spe*I restriction sites, (Figure S1), to give pBinAYA∆CP-3118, pBinAYA∆CP-GFP3444 and pBinAYA∆CP-GFP3609 (the number after GFP represents the predicted size of the cssDNA; Figure 1). These were separately infiltrated into *N. benthamianana* leaves in the presence of pEAQ-*HT*-ACMVCP. The ACMV coat protein was chosen as it has previously been shown to support the formation of larger capsid structures [29]. As expected, green fluorescence under UV light was observed in leaves infiltrated with pBinAYA∆CP-GFP3444 and pBinAYA∆CP-GFP3609 which both contain a full-length copy of the GFP gene that was inserted downstream of the coat protein promoter (Figure 5A). No green fluorescence was evident with construct pBinAYA∆CP-3118 which contains a deleted version of GFP. Virus particles were purified by caesium sulphate gradient centrifugation and fractions analysed by NuPAGE. In the case of particles isolated from leaves co-infiltrated with pBinAYA∆CP-3118 and pEAQ-*HT*-ACMVCP, ACMV coat protein was visible in fractions from the lower part (Fractions 3 to 5) of the gradient when the gel was stained with Instant Blue (Figure 5B left panel, red arrow). When the predicted size of the replicating cssDNA was increased to 3444 or 3609 nucleotides by infiltration with pBinAYA∆CP-GFP3444 or pBinAYA∆CP-GFP3609, no ACMV coat protein could be detected in any fraction by Instant Blue staining, (Figure 5B). However, the ACMV coat protein could be detected by Western blotting using an anti-ACMV serum, in samples from all three co-infiltration experiments, (Figure 5C lower panels, red arrows), indicating the formation of virus particles in all three cases. The fact that Western blotting was needed to detect the coat protein when pBinAYA∆CP-GFP3444 or pBinAYA∆CP-GFP3609 were used for the co-infiltrations, suggests that although particle formation can be achieved upon increasing the encapsidated genome from 2741 to 3118 nucleotides (as in the case of pBinAYA∆CP-3118), assembly is less efficient with these longer cssDNAs. Subsequent transmission electron microscopic examination of a preparation of particles from leaves co-infiltrated with pBinAYA∆CP-3118 and pEAQ-*HT*-ACMVCP (Figure 5D) revealed many particles with differing morphologies, including isometric, double and geminate particles plus a few examples of triple structures (arrowed; Figure 5D). As previously suggested, the mixture of morphologies probably arising through of the lack of coordination between viral DNA synthesis and coat protein expression. Very few particles were observed in the case of infiltration with either pBinAYA∆CP-GFP3444 or pBinAYA∆CP-GFP3609. These data suggest that the supply of the coat protein in trans from a non-replicating vector is not an efficient way of examining encapsidation of longer than genome length cssDNA.

### 3.4. Generation of Triple Geminate Structures Using a Replicating Vector

**Figure 6:**
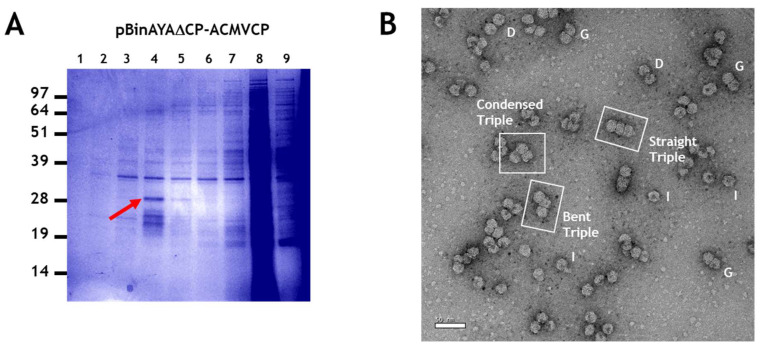
(**A**), Caesium sulphate gradient separation of particles isolated from pBinAYA∆CP-ACMVCP infiltration. Red arrow indicates fraction containing ACMV coat protein. (**B**), Transmission electron microscopy of particles present in the peak fraction, (red arrow) following the infiltration of plant leaves with pBinAYA∆CP-ACMVCP. Isometric (I), geminate (G) and (D) double particles are indicated. The 3 morphological forms of the triple particles—straight, bent and condensed—are indicated. Bar = 50 nm.

To create a more efficient system for examining the encapsidation of greater-than-genome length cssDNA molecules, the ACMV coat protein gene was inserted downstream of the AYVV coat protein promoter in pBinAYA∆CP to create pBinAYA∆CP-ACMVCP. This increased the predicted size of the cssDNA to 3724 nucleotides (Figure 1). This design negated the need to co-infiltrate a coat protein construct since this replicating vector could now directly express the ACMV coat protein. Since the *V2* gene, essential for virus movement [7], was disrupted in this construct, it was incapable of systemic movement and no symptoms developed on the upper leaves of the infiltrated plants by 42 days after infiltration, despite the construct being replication-competent and expressing the ACMV coat protein. NuPAGE analysis of fractions from a caesium sulphate gradient of particles extracted from pBinAYA∆CP-ACMVCP infiltrated leaves (Figure 6A), revealed the presence of ACMV coat protein (arrowed) in fractions 4 and 5 towards the bottom of the gradient. Transmission electron microscopy revealed the presence of many capsid structures within these caesium sulphate fractions. These consisted of isometric, geminate, double particles plus a significant number of triple structures (Figure 6B). In addition, some particles, although conforming to these morphologies, were irregular and readily took up the uranyl acetate stain indicating that they are porous, possibly as a consequence of structural damage. Three distinct morphologies of the triple capsid class can also be discerned, straight, bent or condensed (Figure 6B).

### 3.5. Single Particle Analysis of Triple Particles

**Figure 7:**
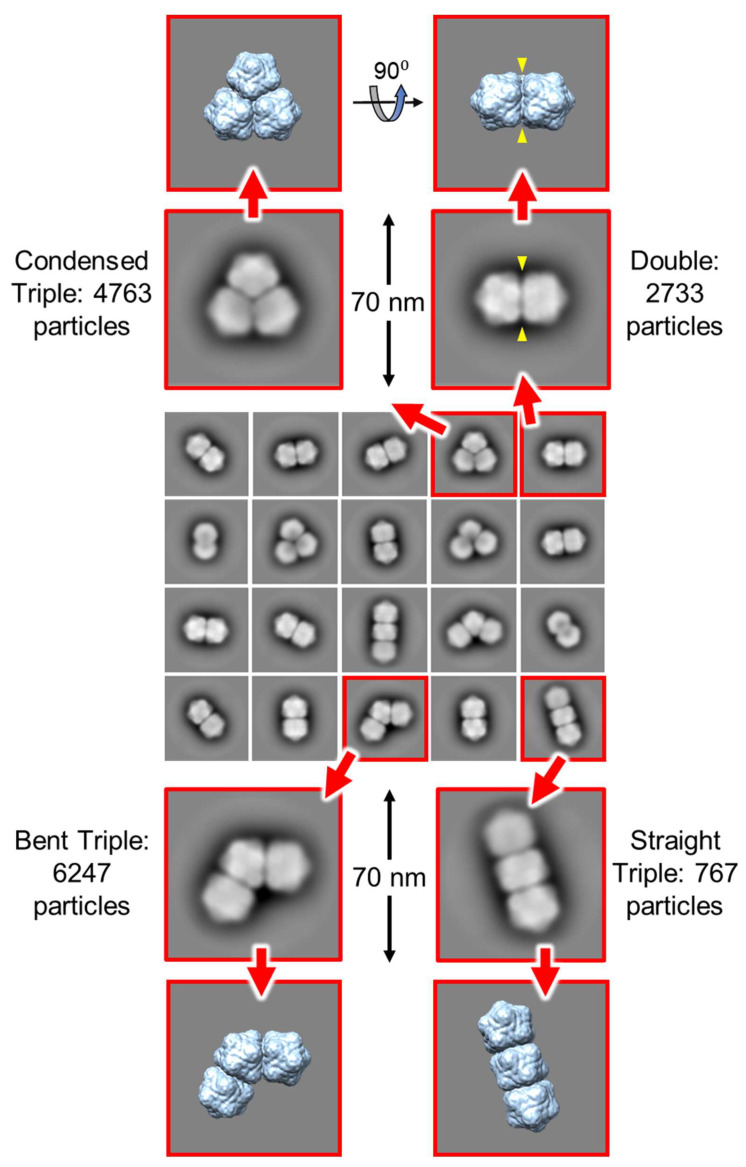
Single particle analysis of the particles imaged from pBinAYA∆CP-ACMVCP infiltration. Several rounds of 2-D classification yielded a particle stack comprised of ~56k particles distributed across 20 classes (central panel). Within these, well-resolved views of condensed, bent and straight Table 3D models (extreme upper and lower panels) are also shown. A comparison between a view perpendicular to the three-fold axis of the condensed triple model (top right) and the classes resembling side views of geminate doubles reveals that the distinctive waist of the latter classes is largely filled in by the third hemisphere lying behind (or in front of) the other two. This suggests that all of these “double” 2-D classes are likely to represent genuine geminate doubles.

**Figure 8:**
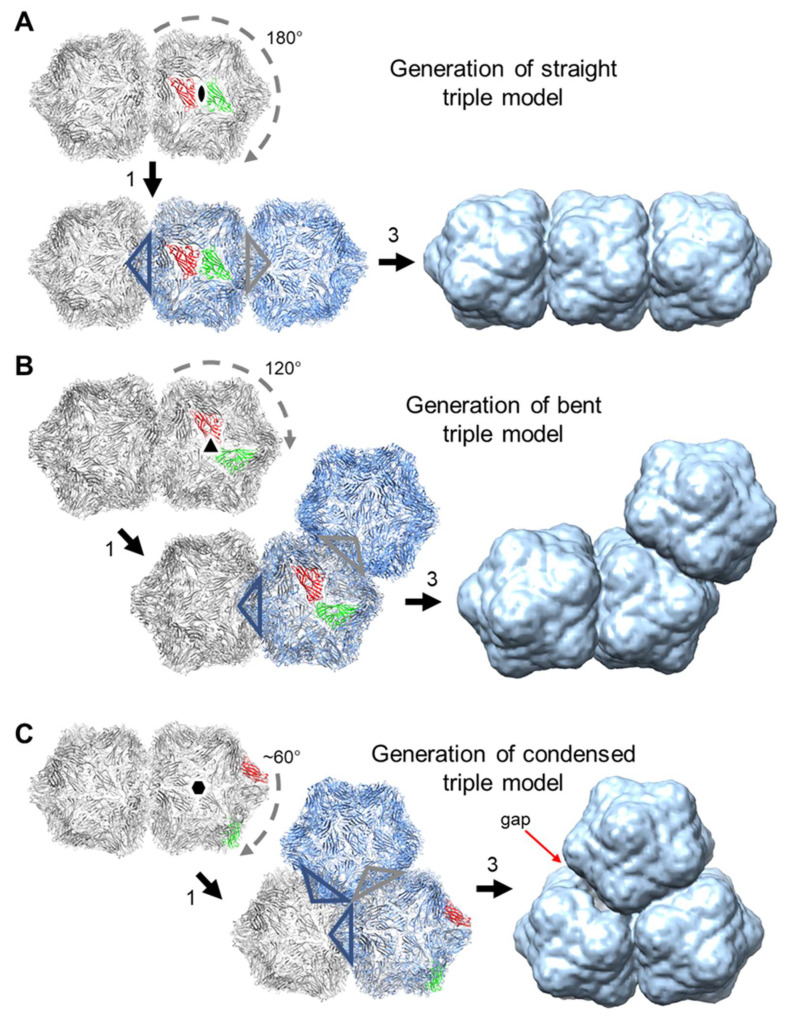
Hypothetical 3-D models of the three morphologies of geminate triples were generated from the previously determined 3.3-Å resolution ageratum yellow vein geminivirus structure (PDB accession code 6F2S; [38]). In each case, the model was created from two copies (distinguished by grey and blue colouration, respectively) of the geminate double structure by superposition based on symmetry-related subunits. Specifically: 1. the red subunit of one copy was aligned with the green subunit of the other copy using UCSF Chimera [51], 2. the two models were merged and one copy of every overlapped pair of subunits was removed as well as all the subunits from the five-fold vertices that were projecting into adjoining partial capsids (the latter are indicated by the colour-coded open triangles; step not explicitly shown in Figure), 3. the resultant models were subsequently rendered as molecular surfaces (not 3-D volumes) at 15 Å resolution to resemble low resolution electron microscope (EM) reconstructions. (**A**), For the straight triple model, the superposition of two-fold related subunits resulted in a 180° rotation of the second copy. (**B**), For the bent triple model, the superposition of three-fold related subunits resulted in a 120° rotation of the second copy. (**C**), For the condensed triple model, the superposition of approximately six-fold related subunits resulted in a ~60° rotation of the second copy. This gave a roughly three-fold symmetric molecular surface with a small gap between two of the partial capsids. By contrast, the corresponding 2-D class average appears to be exactly three-fold symmetric (see Figure 7).

The three morphologies of the triple capsid class were evaluated further by single particle analysis using cryoSPARC. Given the heterogeneous composition of the micrographs, an initial set of reference 2-D templates was generated from manually picked particles in a subset of the images, and these included particles that resembled isometric and geminate double morphologies in projection, but that could represent views of geminate triples where one or two hemispheres are occluded by others. The resultant templates were used to guide auto-picking of the whole dataset, which yielded ~326k particles from the 2466 micrographs. This initial particle stack was contaminated with many false hits, which included overlapping, juxtaposed and broken particles. Several rounds of 2-D classification were used to clean up this stack to give ~56k particles distributed across 20 classes (Figure 7). To aid the analysis of these classes, hypothetical 3-D models for the three geminate triple morphologies were generated [51] from the previously determined 3.3 Å resolution ageratum yellow vein geminivirus structure (PDB accession code 6F2S) [38] as detailed in Figure 8. When appropriately oriented, these models corresponded closely in appearance to the 2-D classes of the three triple morphologies (Figure 7). However, there were no convincing alternative views of triples within these 2-D classes. Indeed, the majority of the well-resolved classes looked like side views of doubles (i.e., observed perpendicular to the long axis), and we speculated that some of these could represent views perpendicular to the three-fold axis of condensed triples. However, a comparison with the corresponding view of the hypothetical condensed model revealed that the distinctive “waist” visible in all the 2-D classes is largely filled in by the third hemisphere lying behind (or in front of) the other two (Figure 7). The dearth of alternative views is not unexpected since the drying step of the negative staining process would tend to force elongated particles to lie flat on the grid and therefore give only a single view. As a result of this preferential orientation, it was not possible generate representative 3-D models from this dataset.

## 4. Discussion

Until recently, studies on the specificity of the interactions between the coat protein and cssDNA of geminiviruses have been hampered by the lack of an assembly system that operates in the absence of a virus infection. Thus, studies on the specificity of cssDNA encapsidation have been limited to indirect methods such as examining how swapping the coat protein between different geminivirus genera affects insect transmission [20], and how less-than-genome length DNAs can be transmitted from plant to plant [46]; the DNA sequences of half-sized *Mastrevirus* DNAs encapsidated within intact isometric particles isolated from infected leaves have been characterized [25]. These were all defective DNAs derived from the genomic sequence and hence all contain exclusively *Mastrevirus*-derived sequences, as opposed to the heterologous sequences of satellites. *Begomovirus* differ from the other genera in being able to efficiently encapsidate such smaller, heterologous sequences. However, the system reported by Hesketh et al. [38] in which a self-replicating alphasatellite cssDNA, approximately half the size of the genomic AYVV DNA, can be encapsidated by transiently expressed AYVV coat protein provides a way of directly examining encapsidation. Using this artificial approach, we have shown that such half-sized molecules can also be encapsidated by the related *Begomovirus,* ACMV, but not by the coat proteins of maize streak virus (*Mastrevirus*) or BCTV (*Curtovirus*). However, when the size of cssDNA was increased to genome size, particles were produced with all four coat proteins. This suggests genome-sized cssDNA molecules are required for stable capsid production in the case of *Curto*- and *Mastre-viruses* but that *Begomovirus* are more flexible in terms of the size of DNA that can be encapsidated. Indeed, particles that resembled one half of a geminate particle, with a flattened surface, were isolated from plants containing defective interfering ACMV DNA [29]. We hypothesise that it is this flexibility that is responsible for the frequent association of smaller DNA molecules, such as the alpha and beta satellite DNAs, defective genomic DNAs and recombinant DNA specifically with *Begomovirus* infections, a phenomenon not generally associated with other geminivirus genera.

Geminiviruses are formed of two capsid hemispheres that are fused together resulting in their distinctive geminate morphology. The N-terminal amino acids of the subunits can adopt different conformations depending on their location in the capsid [24,38,52]. The flexibility of the N-terminus is particularly important at the waist or equatorial region, enabling the two hemispheres to form a tight junction [38]. When critical amino acids in this region are mutated, only isometric capsids are formed on half-length satellite cssDNA [38]. However, doubling the length of the cssDNA results in the formation of particles with two isometric capsids in close proximity to each other when the same mutant coat proteins are expressed. The morphology of these is not geminate, but simply a pair of isometric capsids that here we refer to as “doubles”. These are not stable and readily become independent isometric particles once the cssDNA bridge that is holding them together is broken. The formation and structure of “doubles” suggests that initiation of encapsidation has started at two positions of the cssDNA, potentially opposite each other, rather than at a single equatorial position as previously proposed for the true geminate particles [38,52]. Increasing the length of the cssDNA to 3.1–3.7 kb requires the addition of extra hemispheres for encapsidation, giving rise to triple particles in the presence of wild-type coat protein expressed in trans or from a replicating vector, the latter being more efficient. Stability requires that the junction between each hemisphere must be very similar to the one that fuses the two hemispheres of a geminate particle. Thus, the triples observed possess the straight division between the hemispheres and are not composed of three distinct isometric capsids in close proximity. In addition, we observed many isometric particles in our co-infiltration experiments and unstable doubles, or triples could be the source of them. Over-expression of coat protein coupled to the fact that regulated viral replication is disturbed, could well be another reason for the many isometric particles that are seen.

Other viruses have solved the problem of encapsidating larger genomes by increasing the volume of their capsids by incorporating additional copies of the same coat protein in quasi-equivalent positions to increase the geometry of the capsids from, say, *T* = 1 to *T* = 3 [53]. This results in some of the nucleic acid no longer being in contact with the subunits but rather being in the centre of the expanded particle [54]. Encapsidation of increasing lengths of cssDNA seems to follow a different strategy: increasing the number of *T* = 1 modules in the particle as the length of the DNA increases. This preserves the interactions between the genome and the coat protein subunits. We can speculate about the likely composition of the triples based on their appearance and what we know from the geminate structure. The formation of each interface results in the loss of a pentamer from each hemisphere forming the connection, or ten coat protein subunits for each interface. Thus, for the geminate particles, we have two hemispheres (2 × 60 subunits) and one interface, resulting in 110 subunits. For the triples we have three hemispheres (3 × 60 subunits), and for the straight and bent triples we have two interfaces, resulting in 160 subunits. However, for the condensed triple, there is a third interface, resulting in 150 subunits. Figure 8 illustrates hypothetical 3-D models of the three morphologies found in this study. Future cryo-EM investigations will be necessary to confirm this and to determine the factors that control the position of the third hemisphere to create the straight, bent or condensed triple capsids. The *V2* gene, non-functional in our full-sized coat protein-less replicating cssDNA, may play a role in capsid formation and may possibly control whether straight or the other different triple capsid forms are produced when larger cssDNAs are encapsidated. It is interesting that the vast majority of the triples recorded in SALCV infections of potato were of the straight form [31].

The encapsidated mass of the cssDNA associated with geminivirus infections forms distinct size classes. Why these particular size classes are specifically encapsidated is at present unknown. Betasatellite, alphasatellite and half-length geminivirus cssDNAs are of a similar size. Smaller, deltasatellite DNAs have been characterised in many *Begomovirus* infections [55,56,57] and these are approximately half the size of the alpha and betasatellite cssDNA, or one quarter that of geminivirus cssDNA. Molecular characterisation of plant infections [34,35,36,37] has pointed to another potential group of geminiviruses of a size approximately one and a quarter that of geminivirus cssDNA but their capsid morphology is unknown. We would expect that a genome size of some 4.2-kb nucleotides (one and half that of geminivirus cssDNA) might form quadruple capsid structures. The fact that geminate structures can be formed with alphasatellite DNA [38] suggests that either two molecules of the satellite DNA were encapsidated or that only one copy of the satellite DNA is necessary for the formation of geminate particles. The prevalence of deltasatellite cssDNA in some *Begomovirus* disease complexes suggests that cssDNA of this size maybe sufficient for the formation of stable geminate capsid structures. Further cryo-electron microscopic investigations may well resolve these issues. Conversely, incorporating longer sequences than have been presently tested might lead to the formation of larger structures, such as quadruple particles.

## Supplementary Materials

The following are available online at https://www.mdpi.com/1999-4915/12/11/1235/s1, Table S1. DNA sequences with restriction sites indicated of the oligonucleotides used in this study. Figure S1. (A), Approximate location of PCR primers (Table S1) on pEAQ-*HT*-GFP used to generate DNA fragments for cloning into pBinAYA∆CP. (B), Restriction enzyme cloning sites (*Spe*I and *Age*I) of pBinAYA∆CP. (C), Representation of the resulting cloned DNA fragments in pBinAYA∆CP. The increased size of the resulting replicating virus vectors (pBinAYA∆CP-GFP3444, pBinAYA∆CP-GFP3609 and pBinAYA∆CP-3118 is indicated on the right). Green bar = GFP. Figure S2. MALDI-TOF analysis of the coat proteins of (A), AYVV; (B), ACMV; (C), BeYDV and (D), BCTV derived from the peak fractions of the caesium sulphate gradient separations of Figure 3A.
