# Supplementary material for: Requirements for the Packaging of Geminivirus Circular Single-Stranded DNA: Effect of DNA Length and Coat Protein Sequence
Source: Viruses. 2020 Oct 30;12(11):1235. doi: 10.3390/v12111235 (PMC7694086; doi:10.3390/v12111235)
Supplement: Supplementary file 1 [file viruses-12-01235-s001.pdf]

Table S1. DNA sequences, with restriction sites indicated, of the oligonucleotides used in this study

**KS133P**	*Spe*I *Age*I<br>5'ATTTAATTTCAGTAATTCGTGCTCGCAACTAGTTCGAAGCGACCGGTAGATATTGT CATTTCCACTCCC 3'
**KS134P**	*Spe*I *Age*I<br>3'TAAATTAAAGTCATTAAGCACGAGCGTTGATCAAGCTTCGCTGGCCATCTATAACA GTAAAGGTGAGGG 5'
**KS155**	*Age*I<br>5'TATATACCGGTTTTAAATATGTCGAAGCGACCAGGAGATATCATC
**KS156**	*Xho*I<br>5'AATATACTCGAGTCAATGTTTATTAATTGCCAATACTGTCATAGAAG
**KS153**	*Age*I<br>5'TATATACCGGTTTTAAATATGTCTACTGTGACATGGGGTAAGAAGC
**KS154**	*Xho*I<br>5'AATATACTCGAGATTTTATTGATTTCCAACTGACTTGAAGTACACTC
**KS147**	*Bsp*EI<br>5'AATTCGCGTCCGGATTTAAATATGAGGAAATATACAAGAAATACG
**KS148**	XhoI<br>5'TATATATACTCGAGTTATTAATAAAAATAGCATCTACAATTGCC
**KS138**	XhoI<br>5'TATATATACTCGAGTTATTAATAAAAATAGCATCTACAATTGCC
**KS143**	*Age*I<br>5'CGTACGTAACCGGTTTATTTGTATAGTTCATCCATGCC
**KS144**	*Spe*I<br>5'CGTACGTAACTAGTCAACGTTGTCAGATCGTGCTTCGGCACC
**KS145**	*Age*I<br>5'ACTGACTGACCGGTCTGAAGGGACGACCTGCTAAACAGGAG
**KS174**	*Bsp*EI<br>5' ACTGACTGTCCGGACTGAAGGGACGACCTGCTAAACAGGAG
**KS175**	*Nhe*I<br>5' ACTGACGTGCTAGCTTCTGTATATTCTGCCCAAATTCGCGACCGG

Figure S1. Approximate location of PCR primers on pEAQ-*HT*-GFP, restriction enzyme cloning sites in pBinAYAΔCP and cloned DNA fragments in pBinAYAΔCP.

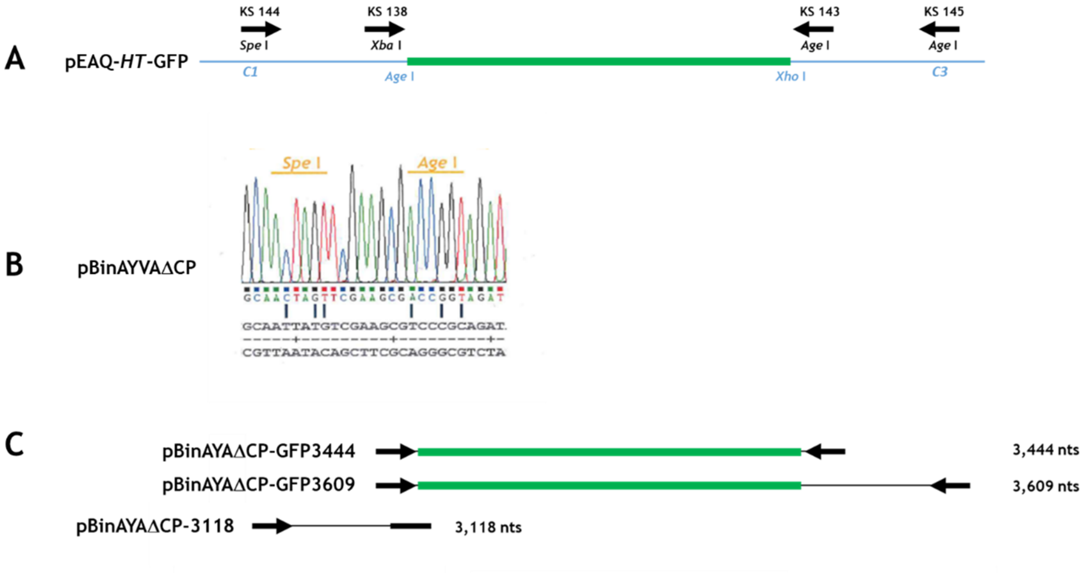

Figure S2. MALDI-TOF analysis of the coat proteins used in this study.

A

KS1 (100%), 29,857.3 Da
AYVV CP 20190228
11 exclusive unique peptides, 22 exclusive unique spectra, 39 total spectra, 134/257 amino acids (52% coverage)

```
MSKRPADIVI STPASKVRRR LNFDTPVMSR AAAPTVLVTN RRRTWTNRPM
YRKPRLYRMY RTPDVPKGCE GPCKVQSYEQ RHDISHVGKV LCVSDVTRGN
GLTHRVGKRF CVKSVYVLGK IWMDENIKTK NHTNTVMFYL VRDRRPFGTA
MDFGQVFNMY DNEPSTATIK NDLRDRYQVL RKFTSTVTGG QYASKEQALV
KKFMKINNYV VYNHQEAAKY DNHTENALLL YMACTHASNP VYATLKIRIY
FYDSVQN
```

B

KS2 (100%), 30,192.6 Da
ACMV CP 20190228
13 exclusive unique peptides, 28 exclusive unique spectra, 34 total spectra, 144/258 amino acids (56% coverage)

```
MSKRPGDIII STPGSKVRRR LNFDSPYRNR ATAPTVHVTN RKRAWVNRPM
YRKPTMYRMY RSPDIPRGCE GPCKVQSFEQ RDDVKHLGIC KVISDVTRGP
GLTHRVGKRF CIKSIYILGK IWLDETIKKQ NHTNNVIFYL LRDRRPYGNA
PQDFGQIFNM FDNEPSTATI KNDLRDRFQV LRKFHATVVG GLYCMKEQAL
VKRFYRLNHH VTYNHQEAGK YENHTENALL LYMACTHASN PVYATLKIRI
YFYDSIGN
```

C

KS3 (100%), 27,056.4 Da
BeYMV CP 20190228
13 exclusive unique peptides, 19 exclusive unique spectra, 24 total spectra, 131/245 amino acids (53% coverage)

```
MSTVTWGKKR KRSGGDSKAK SRGSSSYIPR SVSSRRESLQ VATFSWTSSG
SGIKFSPGGA AYLVSNFPQG ANDNCRHTNK TVLYKFMAKN TIYLDSSHYM
KVFRAPFHFW LVYDKSPGAN VPSTGDIFEG PSLFPHNPWT WTVSRAACHR
FVVKKTWSCV VESNGIDPTK GQGATYYGPG PCNQVKSCNK FFKRLGVSTE
WKNSATGDVG DIKEGALYIV GAPSQKSDVY VNGYFRVYFK SVGNQ
```

D

KS4 (100%), 29,608.0 Da
BCTV CP 20190228
6 exclusive unique peptides, 6 exclusive unique spectra, 14 total spectra, 103/254 amino acids (41% coverage)

```
MRKYTRNTYT MSQKRKVNPQ SAWPKKRRTS TTSRKYQWRR PVTKNRTLKL
KMYDDMLGAG GIGSTISNNG MITMLNNYVQ GIGDSQRAKN VTVTKHLKFD
MALMGSSQFW ETPNYMTQYH WIIIDKDVGS VFPTKLSSIF DIPDNGQAMP
STYRIRRDMN ERFIGKKKWR THWMSTGTGY GGKETYKAPS MPNYKKPMNI
NVRNLNMRTI WKDTGGGKYE DVKENALLYV VVNDNTDNTN MYATLFGNCR
CYFY
```
